# Takotsubo cardiomyopathy: serious early complications and two-year mortality – a 101 case study

**DOI:** 10.1007/s12471-016-0857-z

**Published:** 2016-07-11

**Authors:** M. Zalewska-Adamiec, H. Bachorzewska-Gajewska, A. Tomaszuk-Kazberuk, K. Nowak, P. Drozdowski, J. Bychowski, R. Krynicki, W. J. Musial, S. Dobrzycki

**Affiliations:** 1 Department of Invasive Cardiology, Medical University of Bialystok, Białystok, Poland; 2 Department of Cardiology, Medical University of Bialystok, Białystok, Poland; 3 Department of Cardiology, ProCardia in Augustow, Augustów, Poland; 4 Department of Cardiology, Provincial Hospital in Bialystok, Białystok, Poland; 5 Department of Cardiology, Provincial Hospital in Lomza, Łomża, Poland

**Keywords:** Takotsubo cardiomyopathy, Anterior myocardial infarction

## Abstract

**Background:**

Takotsubo cardiomyopathy (TTC) is characterised by transient contractility disturbances of the apex of the left ventricle.

**Methods:**

We enrolled 101 patients from the northern-eastern part of Poland in the years 2008–2012 who were hospitalised for TCC. The control group consisted of female patients diagnosed with anterior myocardial infarction with ST-segment elevation (anterior STEMI) (*n* = 101).

**Results:**

89 % of the study group were women. Patients with TTC had diabetes (12.6 % vs 29.7 %; *p* = 0.002) and hyperlipidaemia (36.8 % vs 64.4 %; *p* = 0.0001) significantly less frequently, and better kidney function assessed by estimated glomerular filtration rate versus patients with anterior STEMI (74.52 % vs 64.30 %; *p* = 0.004). In the TTC group there were more patients with chronic obstructive pulmonary disease (11.6 % vs 1.0 %; *p* = 0.002) and thyroid disturbances, especially hyperthyroidism (23.4 % vs 11.0 %; *p* = 0.021). In patients with TTC sudden cardiac arrest, pulmonary oedema and cardiogenic shock were observed less frequently than in the control group (14.7 % vs 30.7 %; *p* = 0.0078). Hospitalisations in TTC patients were less frequently complicated by pneumonia (20.0 % vs 35.6 %; *p* = 0.0148) and urinary infection (4.2 % vs 21.8 %; *p* = 0.0003). Cardiac rupture occurred in 3 patients with TTC and in 1 with anterior STEMI. In-hospital mortality was significantly lower in the group with TTC. Also, mortality at 30 days, 3 months, 1 year and 2.5 years was significantly lower in patients with TTC than in patients with MI (*p* = 0.035; *p* = 0.0226; *p* = 0.0075; *p* = 0.009).

**Conclusions:**

Previously considered to be a benign syndrome, TTC should be reconsidered as a clinical condition at risk for serious complications such as cardiac arrest, cardiogenic shock, pulmonary oedema and cardiac rupture leading to death and causing substantial early hazard. The prognosis in TTC is significantly better than in patients with anterior STEMI.

## Introduction

Takotsubo cardiomyopathy (TTC), also called ‘apical ballooning syndrome’ is a clinical condition, in which there are transient contractility disturbances of the apex of the left ventricle and ischaemic changes on the electrocardiogram (ECG) without significant luminal narrowing of the coronary arteries [1–3].

TTC was described for the first time by Sato et al. [1] in Japan in 1990. The name of the syndrome comes from a vessel with a narrow upper part and broad bottom used for catching octopus. This vessel resembles the shape of the left ventricle, as seen on ventriculography in patients with ‘apical ballooning syndrome’ [2].

TTC usually appears in women aged 60–80 years with several cardiovascular risk factors. The syndrome is usually provoked by either mental or physical stress, which is why it is also called ‘stress cardiomyopathy’.

The clinical course of TTC is similar to myocardial infarction and accounts for 1 % of all cases of acute coronary syndromes (ACS). The main symptom is usually chest pain. In laboratory tests an increased concentration of cardiac necrotic markers is observed. On ECG ischaemic changes are recorded [2–6]. TTC usually has a benign clinical course but some cases can be complicated by cardiogenic shock, heart failure, dangerous ventricular arrhythmias or even cardiac rupture and death [7–11].

The aim of the study was to assess the clinical course, comorbidities, complications, early and late mortality in a population with TTC as compared with patients with anterior myocardial infarction.

## Material and methods

In the years 2008–2012, we enrolled 101 patients from the northern-eastern part of Poland hospitalised due to TTC in the Department of Cardiology of the University Hospital in Bialystok and four other cardiology invasive centres (Department of Invasive Cardiology, Medical University of Bialystok; Department of Cardiology, ProCardia in Augustow; Department of Cardiology, Provincial Hospital in Bialystok; Department of Cardiology, Provincial Hospital in Lomza).

Demographic data, cardiovascular risk factors, clinical course during hospitalisation, results of laboratory tests, echocardiography and coronary angiography results were recorded. Only patients with TTC according to the Mayo Clinic criteria were included in the analysis (all four criteria must had been fulfilled):

- Transient hypokinesis, akinesis or dyskinesis of the left ventricular mid segments with or without apical involvement. The regional wall motion abnormalities typically extend beyond a single epicardial coronary distribution. A stressful trigger is often but not always present.
- Absence of obstructive coronary disease or angiographic evidence of acute plaque rupture.
- New electrocardiographic abnormalities (either ST-segment elevation and/or T‑wave inversion) or modest elevation in cardiac troponin.
- Absence of pheochromocytoma or myocarditis. [12]

Five patients were excluded from the analysis, because they were diagnosed with TTC despite significant narrowing in the coronary vessels and one patient due to a muscle bridge in the left anterior descending coronary artery (LAD) with 95 % narrowing during systole.

Obstructive coronary artery disease (CAD) was defined as a narrowing of ≥50 % of the diameter of the vessel. Non-obstructive CAD was defined as any narrowing <50 %.

The control group consisted with female patients diagnosed with acute anterior myocardial infarction with ST-segment elevation (anterior STEMI) hospitalised in the Department of Invasive Cardiology, Medical University of Bialystok in the years 2008–2012 (*n* = 101). The control group matched the study population regarding age and sex.

Anterior STEMI was diagnosed according to the following criteria:

- Clinical symptoms such as chest pain,
- Elevation of cardiac troponin concentration (>0.3 ng/ml),
- ST elevation at the J point in two contiguous anterior leads ( ≥0.1 mV in V1 and V4–V6; ≥0.15 mV in V2–V3).

The primary endpoint was all-cause mortality in a 2-year follow-up. All data were obtained from the Polish population registry in Bialystok (Podlasie Province Office) or by telephone contact with the patients. The study protocol conformed with the ethical guidelines of the 1975 Declaration of Helsinki, and was approved by the local ethics committee (no R‑I-002/26/2013).

## Statistical analysis

Distribution of each variable was tested with the Kolmogorov-Smirnov test. Afterwards the Student’s t test or the Mann-Whitney U test were used for statistical analysis where applicable. Additional analysis of correlations between non-categorical variables was performed using Pearson or Spearman tests, where applicable. Survival rates were displayed with Kaplan-Meier curves. Multivariate logistic regression was used to test associations between variables and outcomes. Data are expressed as means and standard deviations. Relative frequencies are used to present categorical variables. These variables were assessed by the χ^2^ test. A *p* value of less than 0.05 was considered to be statistically significant. The statistic software NCSS 2010 was used.

## Results

Demographic characteristics of the population are shown in Table. 1; 89 % of the study group were women. Patients with TTC had diabetes (12.6 % vs 29.7 %; *p* = 0.002) and hyperlipidaemia (36.8 % vs 64.4 %; *p* = 0.0001) significantly less frequently, and a better kidney function assessed by estimated glomerular filtration rate (eGFR) as compared with patients with anterior STEMI (74.52 vs 64.30; *p* = 0.004) (Table 1, respectively).

**Table 1:** Clinical characteristics, concomitant diseases, clinical course and complications

	TTC *n* = 95 (SD)	Anterior STEMI *n* = 101 (SD)	*p*
**Clinical characteristics**			
– Age (years)	67.6 (14.2)	72.1 (13.1)	**0.023**
– Female sex	89.5 %	100 %	–
– Body mass index, kg/m^2^	25.8 (4.9)	26.5 (6.0)	0.423
– Previous myocardial infarction	4.2 %	8.0 %	0.271
– History of hypertension	63.2 %	68.3 %	0.447
– Diabetes	12.6 %	29.7 %	**0.002**
– Hyperlipidaemia	36.8 %	64.4 %	**0.0001**
– Smoking	21 %	26.3 %	0.383
– Family history of CAD	16.8 %	18.2 %	0.806
**Concomitant diseases**			
– Previous stroke	3.2 %	8.9 %	0.097
– History of malignancy	4.2 %	3.0 %	0.651
– COPD	11.6 %	1.0 %	**0.002**
– Anxiety/depression	8.4 %	6 %	0.515
– Thyroid disorders	23.4 %	11.0 %	**0.021**
Hypothyroidism	8.5 %	7.0 %	0.694
Hyperthyroidism	10.6 %	3.0 %	**0.0335**
Goitre in euthyreosis	4.3 %	1.0 %	0.147
**Symptoms on admission**			
– Retrosternal chest pain	83.2 %	96.7 %	**0.0015**
– Dyspnoea	10.5 %	9.9 %	0.889
– Systolic BP (mmHg)	128.22 (25.99)	133.78 (26.65)	0.141
– Diastolic BP (mmHg)	77.13 (15.29)	82.47 (15.24)	**0.015**
– Heart rate on admission	83.75 (18.55)	88.10 (17.76)	0.095
– Hearty rate on discharge	69.61 (12.34)	80.92 (18.00)	**<0.0001**
– Sudden cardiac arrest	5.3 %	19.8 %	**0.0023**
– Pulmonary oedema	6.3 %	14.85 %	0.0535
– Cardiogenic shock	5.3 %	14.85 %	**0.0267**
– Cardiac arrest, pulmonary oedema, cardiogenic shock	14.7 %	30.7 %	**0.0078**
**Complications**			
– Pneumonia	20.0 %	35.6 %	**0.0148**
– Respiratory failure	7.4 %	10.9 %	0.393
– Urinary tract infection	4.2 %	21.8 %	**0.0003**
– Rhythm disturbances	10.5 %	12.9 %	0.381
Paroxysmal atrial fibrillation	6.3 %	9.9 %	0.357
Ventricular tachycardia	2.1 %	3.0 %	0.690
– AKI/CIAKI	0.0 %	7.9 %	**0.0198**
AKI	0.0 %	3.96 %	0.0508
CIAKI	0.0 %	3.96 %	0.0508
– Cardiac rupture	3.16 %	1.0 %	0.283
– Stroke	0.0 %	4.95 %	**0.0280**
– Hospital psychosis	4.2 %	6.9 %	0.411
In-hospital mortality	3.16 %	9.90 %	0.0581
Discharge home	73.7 %	51.5 %	**0.0014**
Transfer to either hospital, department	23.2 %	40.0 %	**0.0116**
District hospital	17.9 %	33.7 %	**0.0118**
Intensive care unit	5.3 %	6.3 %	0.765
Mean hospitalisation time	5.33 (4.41)	5.41 (2.80)	0.879

*TTC* takotsubo cardiomyopathy, *CAD* coronary artery disease, *COPD* chronic obstructive pulmonary disease, *BP* blood pressure, *AKI* acute kidney insufficiency, *CIAKI* contrast-induced acute kidney insufficiency

In the group with TTC there were more patients with chronic obstructive pulmonary disease (COPD) (11.6 % vs 1.0 %; *p* = 0.002) and thyroid disturbances, especially hyperthyroidism, as compared with the population with anterior STEMI (23.4 % vs 11.0 %; *p* = 0.021). Data on comorbidities are shown in Table 1.

The main symptom in patients with TTC was retrosternal pain, but this was less frequent than in the population with anterior STEMI (83.2 % vs 96.7 %. *p* = 0.0015). In patients with TTC, sudden cardiac arrest, pulmonary oedema and cardiogenic shock were observed less frequently than in the control group (14.7 % vs 30.7 %; *p* = 0.0078) (Table 1).

## Ischaemic changes on ECG

In 55 % of patients with TTC, ST-segment elevation was present on admission to hospital, while in almost every third patient T‑wave inversion was recorded. During hospitalisation, deep negative T waves were observed in 92 % of the population with TTC. Also, a significantly prolonged QTc interval was recorded on the 5th day of hospitalisation in patients with TTC as compared with patients with anterior STEMI.

In the population with TTC there were fewer episodes of atrial fibrillation (5.3 % vs 13.9 %; *p* = 0.042). Other data on cardiac arrhythmias and conductibility disturbances are shown in Table 2.

**Table 2:** Electrocardiogram, echocardiography, angiography and laboratory parameters

	TTC *n* = 95 (SD)	Anterior STEMI *n* = 101 (SD)	*p*
**Electrocardiogram**			
– Rhythm on admission			
Sinus rhythm	91.5 %	86.1 %	0.232
Atrial fibrillation	5.3 %	13.9 %	**0.042**
Pacemaker rhythm	3.2 %	0.0 %	0.084
– Conduction disorders	17.9 %	24.8 %	0.240
LBBB/RBBB	6.3 %	9.92 %	0.355
LBBB	4.2 %	2.0 %	0.372
RBBB	2.1 %	7.92 %	0.064
Left anterior hemiblock	8.4 %	10.9 %	0.554
First-degree atrioventricular block	3.2 %	4.95 %	0.537
– ST elevation	54.7 %	100 %	**<0.0001**
– ST depression	4.2 %	2.0 %	0.372
– T-wave inversion	23.2 %	2.0 %	**<0.0001**
– Deep T‑wave inversion (after a few days)	92.2 %	66.3 %	**<0.0001**
– QT on admission (ms)	406.7 (50.08)	395.04 (45.16)	0.156
– QTc on admission (ms)	468.86 (38.66)	465.59 (28.07)	0.561
– QT after a few days	449.72 (57.73)	414.45 (48.48)	**0.0002**
– QTc after a few days	476.73 (84.49)	469.87 (30.85)	0.494
**Echocardiography/ventriculography**			
– Left ventricular ejection fraction (%)			
EF on admission (%)	39.9 (9.9)	35.1 (9.8)	**0.001**
EF after a few days – mean 4 (%)	48.8 (9.9)	–	**–**
– Left ventricular diameter on admission (mm)	47.6 (4.8)	45.8 (5.2)	**0.014**
– Apical variant of TTC	99 %	–	**–**
– Midventricular variant of TTC	1.05 %	–	**–**
**Coronarography**			
No atherosclerotic changes in coronary arteries	44.2 %	–	–
Insignificant stenosis	55.8 %	3.0 %^a^	–
Significant stenosis	–	95 %^a^	–
**Laboratory parameters**			
– Haemoglobin (mg/dl)	13.35 (1.63)	13.10 (1.35)	0.236
– Erythrocytes (mln/μl)	4.49 (0.65)	4.40 (3.68)	0.263
– Haematocrit (%)	39.54 (6.13)	39.36 (3.68)	0.808
– Leukocytes (thousands/μl)	9.73 (3.96)	12.17 (4.18)	**<0.0001**
– Platelets (thousands/μl)	245.59 (93.32)	243.73 (76.77)	0.055
– Creatinine (mg/dl)	0.90 (0.37)	1.02 (0.65)	0.121
– eGFR MDRD	74.52 (27.21)	64.30 (22.28)	**0.004**
– ASAT (IU/l)	48.79 (35.13)	80.01 (107.71)	**0.027**
– ALAT (IU/l)	31.48 (34.26)	39.94 (55.49)	0.277
– Creatine kinase (IU/l)	435.62 (940.52)	1966.37 (2378.18)	**<0.0001**
– CK-MB (IU/l)	50.15 (72.39)	280.76 (318.5)	**<0.0001**
– Troponin I, mean concentration (ng/ml)	5.32 (11.66)	27.27 (20.98)	**<0.0001**
– Glycaemia on admission (mg/dl)	126.85 (47.35)	180.83 (87.08)	**<0.0001**
– Total cholesterol (mg/dl)	182.07 (41.27)	209.51 (50.14)	**0.0001**
– Low-density lipoprotein (mg/dl)	110.50 (38.12)	140.40 (46.83)	**<0.0001**
– High-density lipoprotein (mg/dl)	52.35 (17.48)	50.21 (17.94)	0.425
– Triglycerides (mg/dl)	98.71 (52.12)	104.65 (68.29)	0.520
– Fibrinogen (mg/dl)	409.53 (160.08)	462.66 (147.90)	**0.033**

*LBBB* left bundle branch block, *RBBB* right bundle branch block, *TTC* takotsubo cardiomyopathy

^a^In two patients with anterior STEMI, coronary angiography was not carried out and they were treated conservatively due to a long delay between the onset of chest pain and hospital admission

## Coronary angiography

In 44 % of the patients with TTC no atherosclerotic changes were observed; in the remaining 56 % of the population changes were insignificant (Table 2).

## Echocardiography and ventriculography

In 95 patients typical contractility disturbances with apical involvement on echocardiography and ventriculography were recorded. We observed transient akinesis of the left ventricular mid segments without apical involvement in one patient. Mean left ventricular ejection fraction (LVEF) was 39.8 % and was significantly higher than in patients with anterior STEMI (35.09 %, *p* = 0.001). On control echocardiography, performed in 30 % of the patients with TTC, improvement of LVEF was recorded (mean LVEF 48.8 %). Other echocardiographic and angiographic data are shown in Table 2.

## Cardiac necrotic markers and other laboratory parameters

Of the patients with TTC, 87.4 % had a significantly raised concentration of cardiac necrotic markers (troponins) but the increase in concentration of cardiac markers was lower than in the population with anterior STEMI (5.32 vs 27.27; *p* < 0.0001). The population with TTC had also a lower white cell count (9.73 vs 12.17; *p* < 0.0001) and lower concentrations of inflammatory parameters such as fibrinogen (409.53 vs 462.66; *p* = 0.033). Patients with TTC had lower glucose concentrations on admission (126.85 vs 180.83; *p* < 0.0001) and lower concentrations of total cholesterol (182.07 vs 209.51; *p* = 0.0001) and low-density cholesterol (110.50 vs 140.40; *p* < 0.0001). Patients with STEMI had a significantly higher concentration of creatinine and lower eGFR assessed by the MDRD formula (74.52 vs 64.30; *p* = 0.004) (Table 2).

## Clinical course and complications

Hospitalisation in TTC patients was less frequently complicated by pneumonia (20.0 % vs 35.6 %; *p* = 0.0148) and urinary infection (4.2 % vs 21.8 %; *p* = 0.0003). No cases of renal failure and strokes were recorded. Cardiac rupture occurred in three patients with TTC and in one with anterior STEMI.

The majority of the patients with TTC were discharged home. The patients from the control group were more frequently transferred to other wards and district hospitals (17.9 % vs 33.7 %; *p* = 0.0118). Other data on clinical course and complications are shown in Table 1.

Data on pharmacological treatment are shown in Table 3.

**Table 3.** Pharmacological treatment and intra-aortic balloon pump (IABP)

	TTC (%) *n* = 95 (SD)	Anterior STEMI (%) *n* = 101 (SD)	*p*
GP IIb/IIIa inhibitors	–	31,7	–
– Abciximab	–	12.9	–
– Eptifibatide	–	18.8	–
Heparin	91.6	98.0	**0.0419**
– Unfractionated heparin	36.8	57.4	**0.0038**
– Enoxaparin	52.6	40.6	0.092
– Fondaparinux	2.1	0.0	0.146
Nitroglycerin	14.7	31.7	**0.0051**
Clopidogrel	82.1	99	**<0.0001**
Aspirin	97.9	98.0	0.951
Statins	89.5	86.1	0.477
Beta blockers	87.4	89.1	0.705
ACEI/ARB	89.5	82.2	0.144
ACEI	82.1	79.2	0.608
ARB	7.4	3.0	0.163
Calcium blockers	8.4	5.9	0.501
Diuretics	49.5	70.3	**0.0029**
Proton pump inhibitors	74.7	90.1	**0.0045**
Pressor amines	13.7	22.0	0.130
Intra-aortic balloon pump	2.11	7.14	0.097

*ACEI* angiotensin-converting enzyme inhibitor, *ARB* angiotensin receptor blocker, *IABP* intra-aortic balloon pump

## Early and late prognosis

**Fig. 1a–d:**
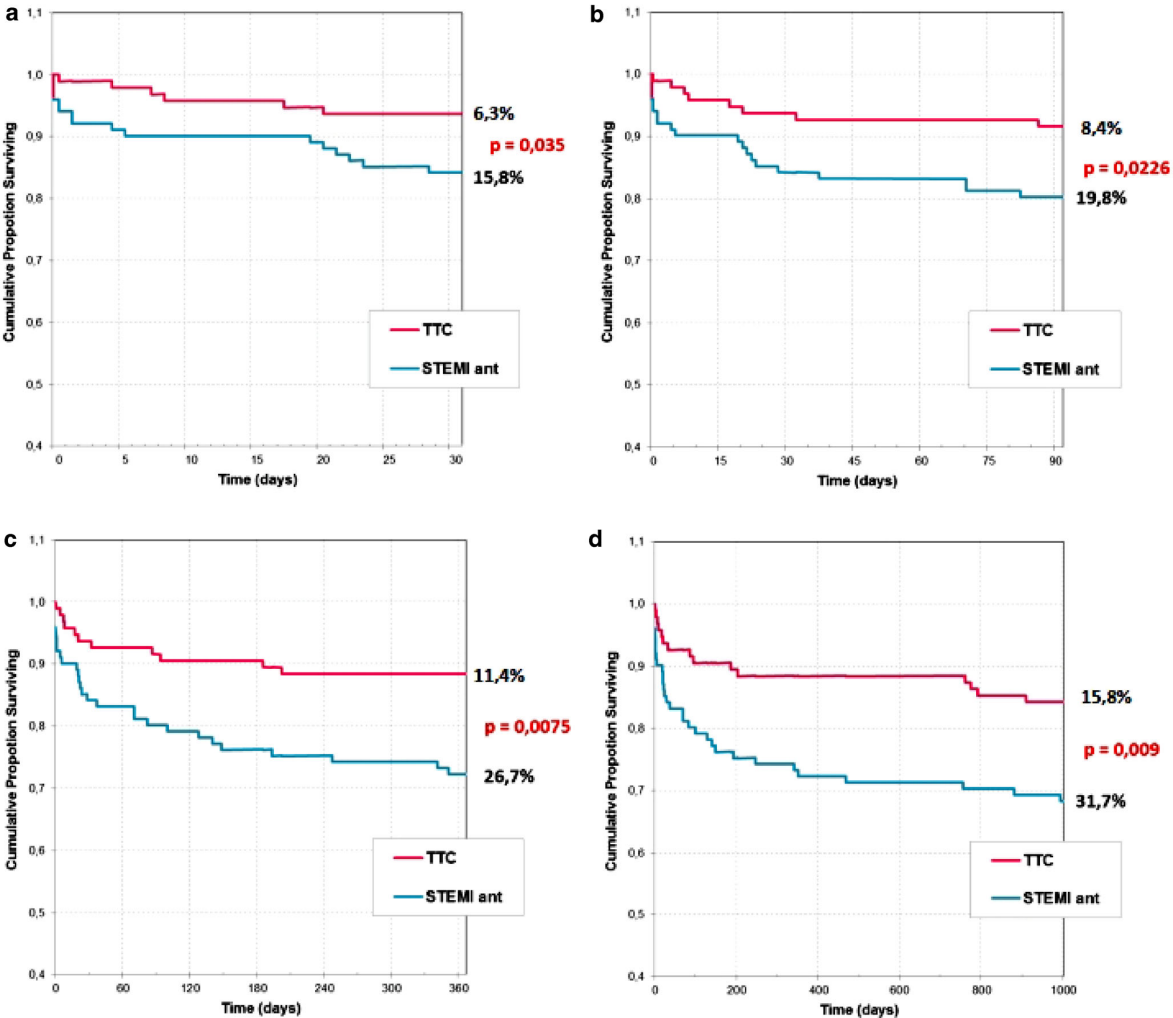
Kaplan-Meier curves showing survival in patients diagnosed with Takotsubo cardiomyopathy and anterior ST-segment elevation myocardial infarction at 30-day, 3‑month, 1‑year and 2.5-year follow-up

In-hospital mortality was significantly lower in the group with TTC. Of three patients who died during hospitalisation, two had a cardiac rupture and one brain injury after a long resuscitation. Mortality at 30 days, 3 months, 1 year and 2.5 years was significantly lower in patients with TTC than in patients with anterior STEMI (*p* = 0.035; *p* = 0.0226; *p* = 0.0075; *p* = 0.009) (Table 1, Fig. 1.).

## Discussion

Over 20 years have passed by since TTC was described by Sato et al. but ‘apical ballooning syndrome’ remains a poorly defined disease entity when it comes to aetiology and management.

In recent years, there were about 2,000 patients per year with MI in the northern-eastern part of Poland (Podlasie Province, data from general cardiology consultant Professor G. Opolski). In the years 2008–2012, 95 patients were diagnosed with TTC in all catheterisation laboratories in Podlasie Province, which accounts for 1 % of all acute coronary syndromes. Stress cardiomyopathy appears mainly in middle-aged women (89 % of our study group). In the similar group with TTC investigated by Tsuchihashi et al. [13] 86 % were women; the mean age was 67. In other published data, women account for 80–100 % of the population with a mean age of 61–76 years [5, 14]. It is worth noting that TTC may also be diagnosed in younger women. In our group the youngest female was 15 years old, the oldest was 100 years.

In our population with TTC, similarly to data from the literature, we observed less cardiovascular risk factors than in patients with anterior STEMI but more cases of COPD (11.6 %) and thyroid disturbances (23.4 %), mainly hyperthyroidism [5].

Disturbances in thyroid function in patients with TTC had already been noticed by other investigators. Sarullo et al. [15] analysed TTC in patients with hyperthyroidism while Cacciotti et al. [16] found hypothyroidism in 29.3 % of their patients with stress cardiomyopathy. The association between hyperthyroidism and hypothyroidism and TTC remains unclear, but many investigators suggest focusing on thyroid function.

In a few papers, COPD was included in the clinical characteristics of the patients with TTC, but only rare single cases were described in which stress cardiomyopathy was linked with COPD as provoking factor. Among other provoking factors, asthma, mental and physical strain were mentioned [13, 17].

The main symptom in TTC was retrosternal chest pain exactly the same as in STEMI. We observed chest pain in 83 % of our patients. In other studies chest pain typical for MI was observed in 54–100 % of the patients with TTC [5].

In our group with TTC we observed serious complications, such as cardiogenic shock, pulmonary oedema and cardiac arrest, significantly less frequently than in patients with anterior STEMI. These adverse cardiovascular events occurred in 14.7 % of the patients with TTC. In other publications cardiogenic shock and pulmonary oedema were observed more often. According to Tsuchihashi et al. [13] pulmonary oedema was observed in 22 % of patients with TTC, and 8 % needed intra-aortic counterpulsation. Surprising data were published by Song et al. [17]. They observed cardiogenic shock in 35 % and pulmonary oedema in 42 % of their patients with TTC. On the other hand Parodi et al. [18] reported cardiogenic shock in 5 % of their population with TTC.

In our study 3 patients (3.16 %) with TTC had cardiac rupture, while in the anterior STEMI group in which there is a high risk of cardiac rupture – only one. In their systematic analysis, Kumar et al. [9] described 12 cases of cardiac rupture in patients with TTC. Ten patients out of 12 died. In the majority of them perforation of the left ventricular wall was confirmed on autopsy. In our study 2 patients died. One female with cardiac rupture in the apex of the left ventricle diagnosed on ventriculography survived thanks to urgent cardiac surgery.

In-hospital and late mortality were significantly lower in the group with TTC than in patients with MI. During hospitalisation, 3 (3.16 %) patients with TTC died due to cardiac complications. Exactly the same 3 % in-hospital mortality due to cardiovascular reasons was reported by Nunez-Gil et al. [19] and 2 % cardiovascular mortality by Parodi et al. [18] In some observational studies there were no deaths in patients with TTC during hospitalisation but according to other authors mortality is much higher and even reached 8 % [20]. After 2.5 years, 15.8 % of our study patients had died. The reasons of death were not connected with cardiovascular reasons but mainly with cancer. Song et al. [17] observed 9 % mortality after 5‑year follow-up. The reasons of death were non-cardiac.

TTC is one of the least known cardiac diseases. Although clinical presentation is similar to MI, the prognosis is much better. Nevertheless it is worth noting that the course of TTC can be complicated by serious events such as cardiac rupture, pulmonary oedema, cardiac arrest which causes substantial early hazard.

### Study limitations

Takotsubo cardiomyopathy was diagnosed according to Mayo Clinic criteria. Of the patients from takotsubo group, 56 % had non-obstructive CAD. We could not exclude spasm and spontaneous thrombolysis in patients with non-obstructive CAD.

## Conclusions

Previously considered to be a benign syndrome, TTC should be reconsidered as a clinical condition at risk of serious complications such as cardiac arrest, cardiogenic shock, pulmonary oedema and cardiac rupture leading to death. The TTC population was diagnosed with a significantly higher rate of COPD and thyroid disorders than patients with STEMI. Early and late prognosis in patients with TTC is better than in patients with anterior STEMI.
